# Multi-dimensional role of gangliosides in modulating cancer hallmarks and their prospects in targeted cancer therapy

**DOI:** 10.3389/fphar.2023.1282572

**Published:** 2023-11-27

**Authors:** Abhisek Sarkar, Sounak Banerjee, Kaushik Biswas

**Affiliations:** Department of Biological Sciences, Bose Institute, Kolkata, India

**Keywords:** cancer, tumor, gangliosides, hallmarks, targeted immunotherapy, anti-ganglioside CAR-T cells

## Abstract

Gangliosides are glycosphingolipids with prevalence in nervous tissue and their involvement in certain neuronal diseases have been widely known. Interestingly, many recent studies highlighted their importance in the development and progression of various cancers through orchestration of multiple attributes of tumorigenesis, i.e., promoting migration, invasion, escaping the host immune system, and influencing other cancer hallmarks. Therefore, the multidimensional role of gangliosides in different cancers has established them as potential cancer targets. However, the tremendous structural complexity and functional heterogeneity are the major challenges in ganglioside research. Moreover, despite numerous immunotherapeutic attempts to target different gangliosides, it has failed to yield consistent results in clinical trials owing to their poor immunogenicity, a broad range of cross-reactivity, severe side effects, lack of uniform expression as well as heterogeneity. The recent identification of selective O-acetylated ganglioside expression in cancer tissues, but not in normal tissues, has strengthened their potential as a better and specific target for treating cancer patients. It was further supported by reduced cross-reactivity and side effects in clinical trials, although poor immunogenicity remains a major concern. Therefore, in addition to characterization and identification of the biological importance of O-acetylated gangliosides, their specific and efficient targeting in cancer through engineered antibodies is an emerging area of glycobiology research. This review highlights the modulatory effect of select gangliosides on different hallmarks of cancer and presents the overall development of ganglioside targeted immunotherapies along with recent progress. Here, we have also discussed its potential for future modifications aimed towards improvement in ganglioside-based cancer therapies.

## 1 Introduction

Gangliosides, sialic acid containing glycosphingolipids, localized in the external leaflet of the plasma membrane, have gained much importance in the recent years for their involvement in manipulating the established and widely recognized Hallmarks of Cancer, apart from their well-known function of maintaining membrane integrity, regulating cellular communication, and transducing cellular signals. Accumulating evidence from recent progress in the study of gangliosides revealed their crucial involvement in various cancers. High structural variation and discrete expression in the different developmental stages as well as in various pathophysiological conditions make them one of the most interesting candidates for further functional exploration.

The structural complexity of gangliosides primarily arises out of a high degree of structural diversity owing to the variations in the arrangement of oligosaccharide moieties, as well as sialic acid residues, which makes them an intriguing yet extremely complex subject to study their biological role and function. Structural analysis of different gangliosides reveals their enormous variation, e.g., ganglioside GM3 and GD3 are structurally separated by only one sialic acid residue whereas, GM1 and GM2 are differentiated by only one galactose moiety. A close structural similarity is noted in the case of ganglioside GM1a and GM1b. Not only the subtle structural changes and positional differences of sialic acids make gangliosides highly heterogeneous but differential acetylation in the sialic acid residues makes them more diverse. O-acetylation of gangliosides is one of the major modifications in the sialic acids and most prevalent in cancer tissues compared to normal tissues (Cavdarli et al., 2020a). Gangliosides carrying O-acetylated sialic acids are often found in developing tissues, especially of neuroectodermal origin and are considered as markers in a variety of tumors but their precise biological function in tumorigenesis is yet to be unfolded properly. A study focused on the identification and expression analysis of O-acetylated gangliosides in melanoma, glioblastoma, and breast cancer cell lines identified the prevalence of different O-acetylated gangliosides, namely, O-Ac-GM1, O-Ac-GD2, O-Ac-GD3, O-Ac-GT2 and O-Ac-GT3. Although, acetylation can occur in the C4/7/8/9 positions within the sialic acid residue, however 9-O-Ac and 8-O-Ac are considered as most stable among the others (Vandamme-Feldhaus and Schauer, 1998). 9-O-Ac-GD3 is the well-studied acetylated variation of the gangliosides among all, which is considered as an onco-fetal marker in tumors with neuroectodermal origin, such as neuroblastoma, and melanoma, or breast cancer. 9-O-Ac-GD3 exhibits anti-apoptotic property unlike GD3. Preincubation of 9-O-Ac-GD3 resists GD3 induced pro-apoptotic activity in Jurkat and Molt-4 cells (Irani et al., 1996). Exogenous administration of 9-O-ac-GD3 prevents mitochondrial membrane depolarization, release of cytochrome C and further caspase activation to prevent GD3 induced apoptosis in lymphoblasts (Kniep et al., 2006; Mukherjee et al., 2008). O-Ac-GD2 is also studied extensively and found to be overexpressed in glioblastoma, unlike GD2, which is more prevalent in normal brain tissues. This exclusivity of O-Ac-gangliosides makes them a suitable target for cancer immunotherapies, which will be discussed later in this review. Though the roles of O-Ac-GD3, and O-Ac-GD2 are well studied, and several clinical studies are ongoing focusing on targeting O-Ac gangliosides in different cancers as an immunotherapeutic measure, but little is known regarding the role of O-Ac-GM1, O-Ac-GT2, and O-Ac-GT3 in tumorigenesis. Aberrant expression of gangliosides in different cancers is associated with not only poor prognosis but also low survival (Cochonneau et al., 2013). For example, O-acetyl-GD3 and 9-O-acetyl-GT3 were reported to be overexpressed in almost 50% of patients with invasive ductal carcinoma (Marquina et al., 1996). N-Glycolyl-GM3 is also detected in almost 100% of stage II breast cancers (Oliva et al., 2006). A study on clear cell type renal cell carcinoma (RCC) patients also reported the over-expression of ganglioside GM2 (Banerjee et al., 2019). Increasing evidence suggests the over-expression of these gangliosides not only points towards its association with poor pathophysiological conditions but also with reduced survival, thereby contributing to a poor prognosis. An extensive study on the functional significance of differential ganglioside expression in various cancers reveals their positive association in promoting invasion (Hamamura et al., 2005), migration (Kundu et al., 2016), and epithelial to mesenchymal transition (EMT) (Vantaku et al., 2019), that together contribute towards an alteration of metastatic progression pattern (Zhang et al., 2006) of the tumor cells. Tumor shed gangliosides in their microenvironment also potentiate cancer cells to escape host immune surveillance by disrupting cell-mediated immune responses (Raval et al., 2007). Interestingly gangliosides are also reported to influence tumorigenesis by differentially regulating cell growth and apoptogenic signaling pathways (Colell et al., 2001). Based on these studies, gangliosides have emerged as a unique group of molecules that have such profound and prominent effects on various hallmarks of cancer. Though the positive association of gangliosides in tumor progression identifies them as potential immunotherapeutic targets, near-structural similarities between different gangliosides make it difficult to target a particular ganglioside without any cross-reactivity. A broad range of cross-reactivity among different gangliosides may contribute to tremendous variation in clinical outcomes. The identification of O-Acetylated gangliosides and their associated expression in tumor tissues compared to their normal counterpart makes them a promising target for the development of future immunotherapeutic strategies. Compared to unmodified gangliosides, these acetylated gangliosides are better suited as target molecules in cancer immune therapies. Targeting these molecules with specially engineered antibodies ensures its higher specificity towards cancer tissues, leaving the normal tissues unharmed, thereby reducing the severity of the side effects associated with the non-specificity of the cancer treatments. Identification and characterization of several O-acetylated gangliosides will provide an immense scope towards developing more specific therapeutic strategies thereby minimizing side effects. This review addresses the intricate involvement of gangliosides in various pathways that identify and regulate the hallmarks of cancer as well as the various therapeutic strategies utilizing gangliosides as potential anti-cancer targets. We also aimed to concentrate on their success, shortcomings, and recent innovations in this field, which show an enormous potential for enhancement.

## 2 Ganglioside biosynthesis and cellular transportation

**FIGURE 1:**
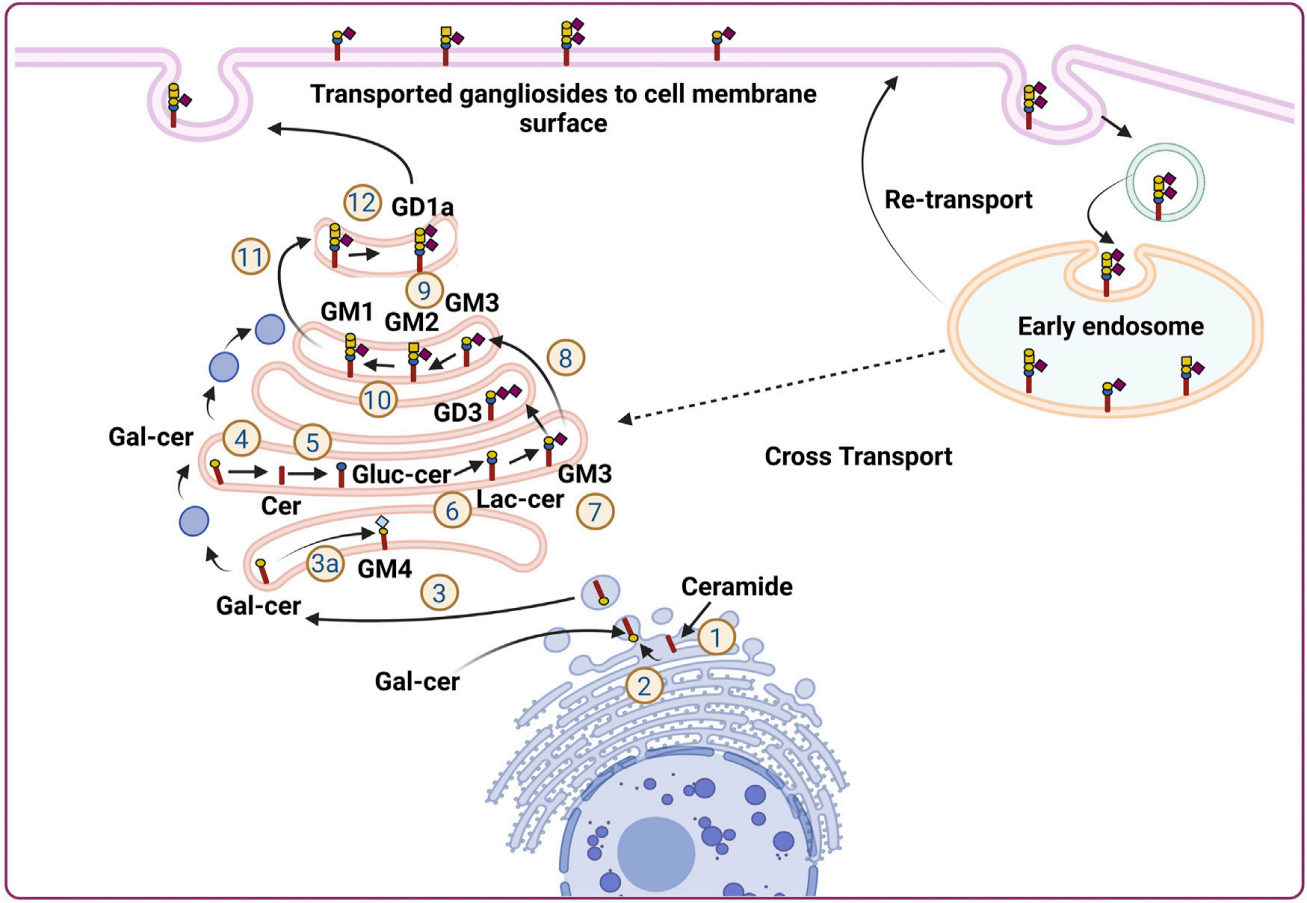
Subcellular localization and transportation of gangliosides in the cells. Ganglioside biosynthesis is initiated in the endoplasmic reticulum (ER) with the synthesis of ceramides (Cavdarli et al., 2020a). Ceramides then converted to glucosylceramide (Glc-cer) in Golgi apparatus (Cavdarli et al., 2020b), which is then converted to lactosylceramide (Lac-cer) and acts as a precursor for all other gangliosides except GM4 (Vandamme-Feldhaus and Schauer, 1998) Lac-cer later modified to generate different gangliosides for, e.g., GM3, GD3, GM2, GM1, GD1a (Irani et al., 1996; Kniep et al., 2006; Mukherjee et al., 2008; Cochonneau et al., 2013). In a separate pathway, within Endoplasmic reticulum, ceramide is converted to galactosylceramide and moved to Golgi apparatus where, it is converted to GM4. The gangliosides generated in the Golgi apparatus are then finally transported to the cellular membrane by vesicular transportation. Furthermore, the ganglioside moiety present on the plasma membrane further, transported back to the endosomal vesicles via endocytic pathway to be integrated into the Golgi apparatus to continue ganglioside metabolism process.

**FIGURE 2:**
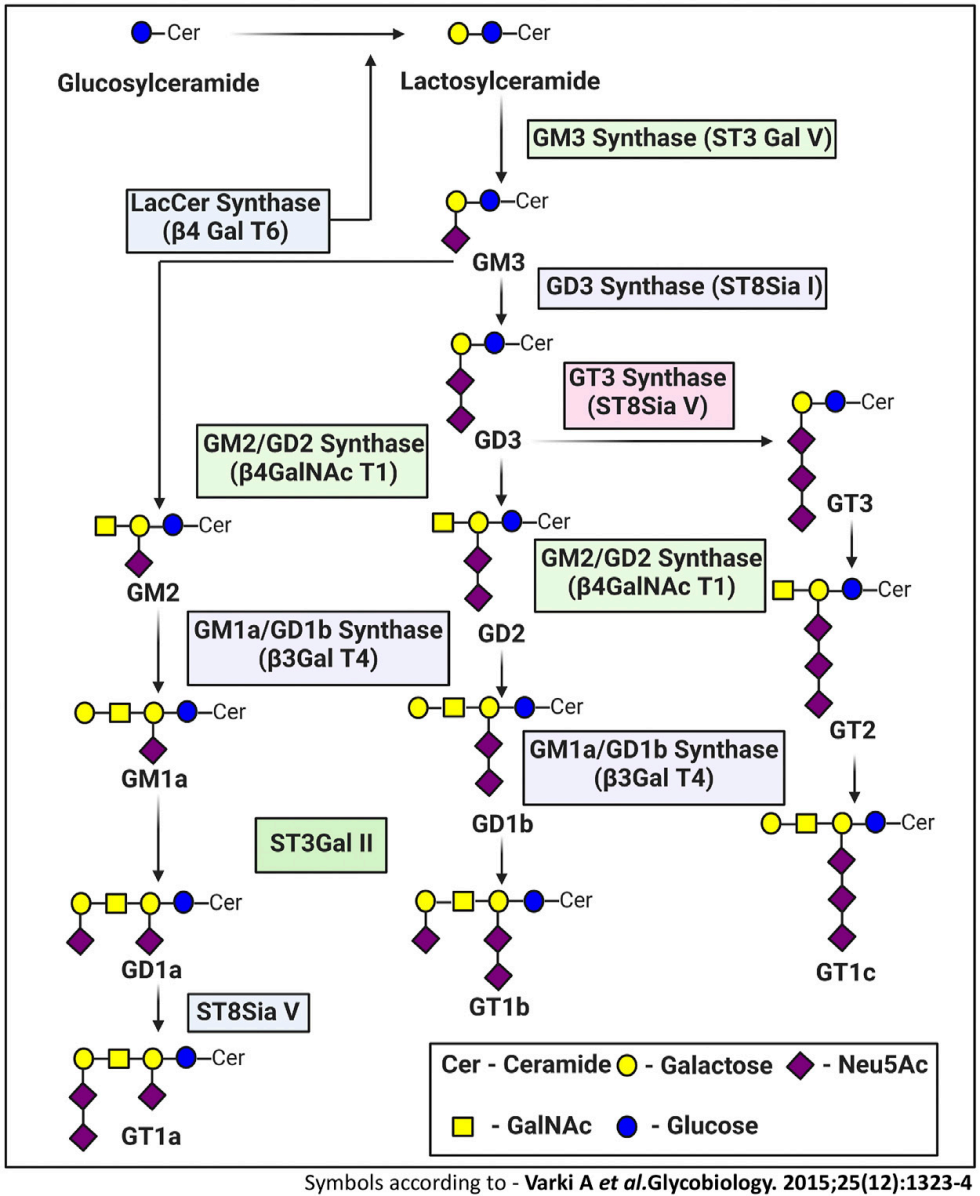
Ganglioside biosynthetic pathway. The *de novo* biosynthesis of ganglioside is initiated by ceramide synthases with the formation of the common precursor, ceramide (Cer). Ceramides are then converted to glucosylceramide (GlcCer). The initial steps of glycosylation include the transfer of glucose or galactose residues to the Cer moiety to produce either glucosylceramide (GlcCer). Initially synthesized glucosylceramides are subjected to further modification by addition of a galactose moiety to form lactosylceramide (LacCer), a common precursor for almost all gangliosides (except GM4). The inclusion of one sialic-acid residue to LacCer then converts this precursor molecule to GM3, catalyzed by sialyltransferase I (ST-I) or GM3 synthase. Further addition of sialic-acid residues catalyzed by sialyltransferase-II or GD3 synthase and sialyltransferase-III or GT3 synthase leads to the generation of GD3 and GT3, respectively. LacCer, GM3, GD3, and GT3 act as precursors for other complex gangliosides of the 0-, a-, b-, and c-series. These precursors undergo sequential glycosylation by b4GalNAcT 1, b3GalT 4, ST3 Gal II, and ST8Sia V to yield more complex gangliosides.

Gangliosides are made up of a core lipid molecule, the ceramide, which is further coupled with a glycan head group of various complexities that contribute to their structural diversity. In cells, gangliosides are mainly localized in the upper leaflet of the plasma membrane accompanying both sphingomyelin and cholesterol, together which form membrane micro-domains (Simons and Sampaio, 2011). These micro-domains play very important roles in receptor positioning, cellular communication, and signal transduction (Lopez and Schnaar, 2009). The *de novo* biosynthesis of gangliosides is initiated with the formation of the common precursor, ceramide (Cer) within the endoplasmic reticulum (ER) by ceramide synthases (Stiban et al., 2010). Ceramides are then moved to the Golgi apparatus either by vesicular transport or aided by Ceramide transport proteins (CERTs) (Hanada et al., 2009). Within the Golgi apparatus, ceramides are converted to glucosylceramide (GlcCer), which is subsequently modified by other carbohydrate moieties, catalyzed by specific glycosyltransferases. The graphical representation of subcellular localization and transportation is depicted in Figure 1. The initial steps of glycosylation include the transfer of glucose or galactose residues to the Cer moiety to produce either glucosylceramide (GlcCer) or galactosylceramide (GalCer), respectively. Initially synthesized glucosylceramide then gets further modified by addition of a galactose moiety to form lactosylceramide (LacCer), a common precursor for almost all gangliosides (except GM4). The addition of a single sialic-acid residue to LacCer then converts this precursor molecule to GM3, catalyzed by sialyltransferase I (ST-I) or GM3 synthase. Subsequently, GD3 and GT3 can be further generated by subsequent addition of sialic-acid residues, catalyzed by sialyltransferase-II or GD3 synthase and sialyltransferase-III or GT3 synthase, respectively. LacCer, GM3, GD3, and GT3 act as precursors for other complex gangliosides of the 0-, a-, b-, and c-series. These precursors undergo a series of glycosylations by β4GalNAcT 1, β3GalT 4, ST3 Gal II, and ST8Sia V to yield more complex gangliosides (Crespo et al., 2010). The overall biosynthetic pathway of ganglioside is depicted in Figure 2.

## 3 Ganglioside and hallmarks of cancer

**FIGURE 3:**
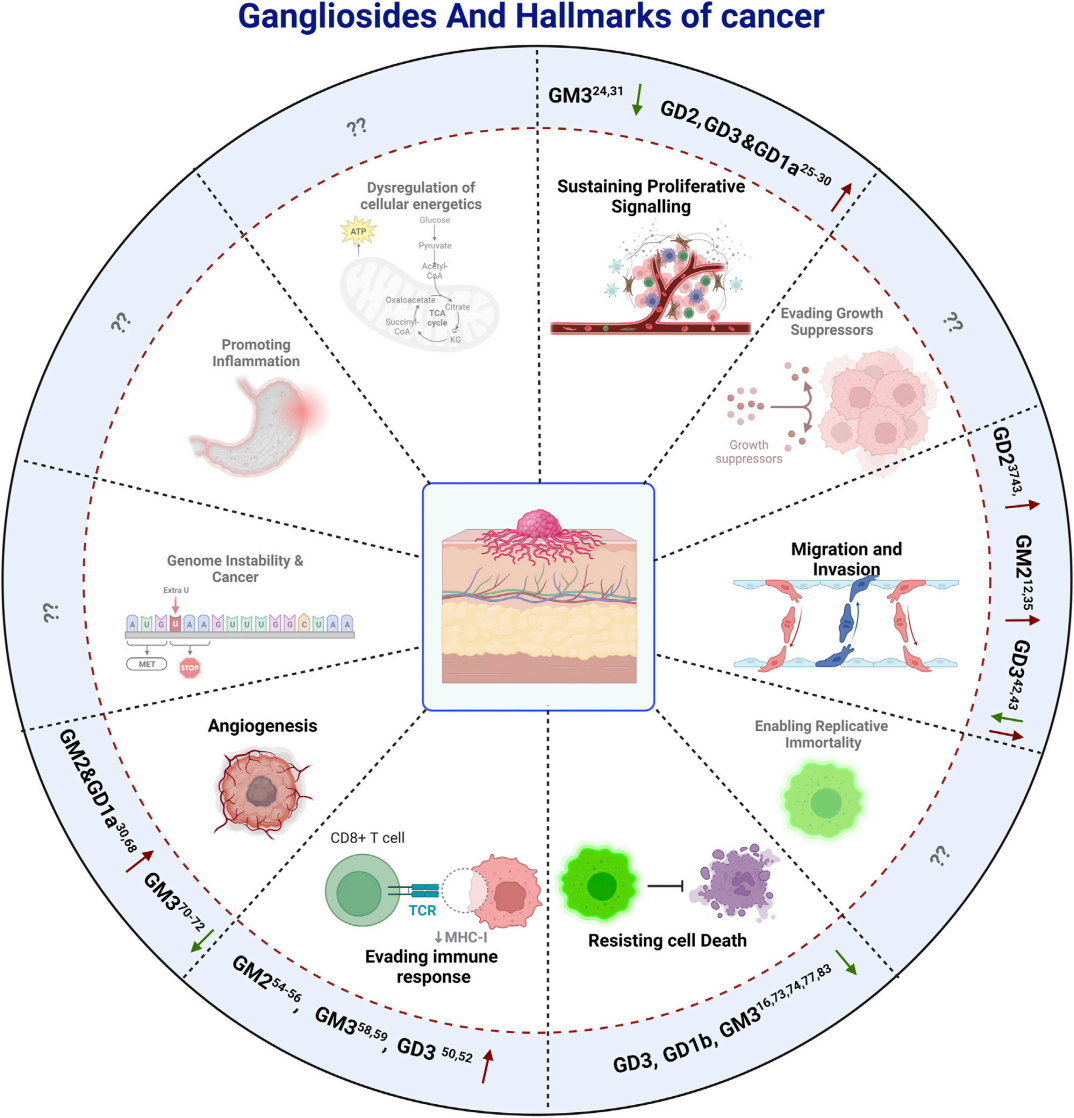
The effect of gangliosides on The Hallmarks Of Cancer. Here positive and negative impacts of different Gangliosides on different hallmarks are depicted by Up and Down arrows respectively, while the hallmarks on which there are no reported information, are depicted by question marks, indicating a potential field of study for future investigations.

This brings us to the Hallmarks of cancer which are considered as unique fundamental characteristics that a cell needs to acquire in order to endow itself with specific abilities that defines a cancer cell. These hallmarks not only help to define the characteristics of cancer cells, but also enable cancer cells to acquire the ability to trick the host system, enabling it to survive, grow and progress. In other words, the demarcation of the hallmarks has provided a perfect framework for understanding and generating new information about cancer and their long-term implications on the host system. As gangliosides are shown to have modulating effects on various aspects of cancer over the past few years, its relationship with the different hallmarks has become more and more evident, and our understanding of this has been constantly evolving. Traditionally the cancer hallmarks were divided into six categories (Hanahan and Weinberg, 2000) before addition of another four (Hanahan and Weinberg, 2011). Understanding how different ganglioside species affect the various hallmarks distinctly to regulate tumorigenesis, will ultimately provide clues to target gangliosides towards a favorable outcome. A graphical representation, illustrating the regulatory role of various gangliosides on different hallmarks of cancer is given in Figure 3.

## 4 Modulating proliferative signaling

**FIGURE 4:**
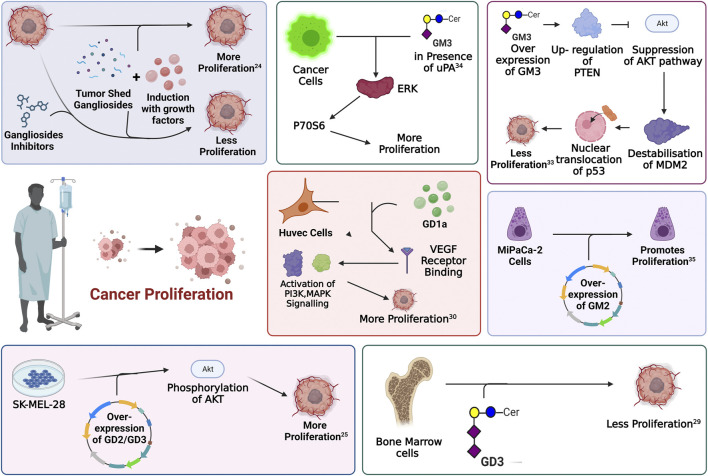
Involvement of gangliosides in the regulation of proliferative signaling. Every enclosed area in the figure represents an information about cellular pathways and their modulation by gangliosides which ultimately affects cellular proliferation.

One of the fundamental processes of tumor progression is cellular proliferation where cancer cells show an alteration of cell cycle, growth-related proteins and signaling pathways that lead to an un-controlled cell-division by passing all the programmed cell death mechanisms. The role of gangliosides in the proliferation of cancer has been extensively studied in the past years. In a pioneering work of Stephan Ladisch, the role of gangliosides, precisely GM3 and GD1a in tumor cell proliferation, was highlighted. This is among the very few studies that identified the involvement of gangliosides in growth factor-induced cellular proliferation. Here, growth factor (bFGF, IGF-I, PDGF) mediated proliferation of mouse embryonic fibroblast cell line Swiss 3T3 was significantly blocked by chemical inhibition of ganglioside synthesis by d-l-threo-1-phenyl-2-hexadecanoylamino-3-pyrrolidino-1-propanol-HCl (PPPP) (Li et al., 2000), indicating a potential pro-proliferative role of certain gangliosides. Human melanoma cell line, SK-MEL-28 N1 with stable overexpression of GD2 and GD3 demonstrated significantly higher proliferative activity, compared to those overexpressing GM2 and GM1 which did not show any significant proliferative activity. This observation was further supported by the increased phosphorylation of Akt at Ser473 and Thr308 in GD2 and GD3 overexpressing cell lines, indicating an activation of Akt signaling pathway. Activation of Akt pathway is associated with increased cellular proliferation, cellular growth and metastasis (Ohmi et al., 2018). Similar overexpression of GD3 synthase gene in human small cell lung cancer cell lines (SCLC) and in breast cancer cell lines, upregulates the expression of GD2 and GD3, markedly increasing the proliferative activity *in vitro*. In SCLC cell line, SK-LC-17 which did not express b-series gangliosides (GD2, GD1b, and GT1b), GD3-synthase was overexpressed to assess the role of b-series gangliosides. GD3-synthase overexpressed cells exhibited an increased proliferation compared to the control cells. It was further observed that overexpression of GD3-synthase was associated with an increased phosphorylation of ERK1, suggesting the activation of proliferative signaling. This increased proliferation was further restricted by incubating the culture medium with anti-GD2 antibody, which indicates the positive role of GD2 in the proliferation of SCLC *in vitro*. In breast cancer cell line MDA-MB-231 stable overexpression of GD3-synthase was associated with increased proliferative activity owing to the activation of MEK/ERK and PI3K/Akt signaling pathway. Further analysis identified the constitutive activation of an upstream element c-Met leading to GD3-synthase dependent proliferative activity in MDA-MB-231 cells (Yoshida et al., 2001; Cazet et al., 2010; Cazet et al., 2012). Furthermore, mouse T cell lymphoma or Dalton’s lymphoma derived gangliosides demonstrate a significant anti-proliferative activity over bone marrow cells (BMCs) both *in vitro* and *in vivo*. Among all the complex gangliosides, GD3 is recognized as one of the prominent gangliosides to block the proliferation of BMCs (Bharti and Singh, 2001). In normal human umbilical vein endothelial cells (HUVEC), GD1a promotes VEGF-dependent proliferative activity. It was suggested that GD1a might facilitate the binding of VEGF with its receptor or stabilizes the ligand-receptor complex to promote cellular proliferation by activating downstream PI3K, MAPK signaling (Lang et al., 2001). Thus, several evidence point towards a context-dependent role of select gangliosides towards cellular proliferation, in connection with tumorigenesis. Exogenous administration of GM3, significantly blocks the proliferative activity of human keratinocytes (Paller et al., 1993), murine bladder cancer cell line such as MBT-2 (Watanabe et al., 2002) and human colon cancer cell lines. It was further identified that overexpression of GM3, upregulated the expression of PTEN followed by the suppression of Akt pathway. Suppression of Akt pathway leads to destabilization of MDM2 and promoted the nuclear translocation of p53 leading to the transcriptional activation of p21WAF1. Activation of p21WAF1 was associated with the cell cycle arrest and inhibited proliferation, in response to GM3 in colon cancer cell line HCT-116 (Choi et al., 2006). A paradoxical role of GM3 was also reported in human squamous carcinoma cell, SCC12F2 where overexpression of GM3 increased the cellular proliferation in the presence of urokinase plasminogen activator protein (uPA). There are several cancer types which show a higher expression of uPA. Increased uPA is associated with activation of uPA mediated signaling cascade leading to enhanced cellular proliferation and promoting tumorigenesis. In squamous cell carcinoma cell line, overexpression of GM3 reorganizes the lipid raft to conglomerate uPARs (urokinase plasminogen activator protein receptor) on the plasma membrane. This explains the observation that, in presence of uPA, overexpression of GM3 leads to the hyper activation of uPA/uPAR signaling axis and enhanced phosphorylation of p70S6 kinase. Increase in p70S6 kinase phosphorylation leads to its activation and promotes cellular proliferation in the presence of GM3 in squamous cell carcinoma cell line (Wang et al., 2006). Although a recent study on the association of overexpressed GM2 with tumorigenesis in pancreatic ductal adenocarcinoma (PDAC) reveals that a subpopulation of PDAC cell line MIAPaCa-2, over-expressing GM2 exhibits a significantly increased proliferative activity (Sasaki et al., 2019). Interestingly other reports indicate that either overexpression, silencing or genomic knockout of GM2-synthase does not affect proliferative capacity of several cancer cell lines (Mahata et al., 2015a; Kundu et al., 2016). Figure 4 illustrates the role of different gangliosides in the differential regulation of cancer cell proliferation.

## 5 Effect on invasion and migration

Tumor development and progression is associated with the characteristic collective migration of the primary tumor to spread distantly throughout the body. Over the recent past years, various studies have revealed pro-migratory and pro-invasive roles of various gangliosides which have direct implication in the development of cancer. Ganglioside GD2, which is overexpressed in several cancer cells and tissues of neuroectodermal origin is associated with high metastatic and invasive abilities. In a study with experimentally transformed human mammary epithelial cells (HMECs) with oncogenic V12-H-Ras mutations (HMLER cells), a subset of cells showed a striking overexpression of ganglioside GD2. Upon further analysis, these GD2 overexpressing subsets showed greater migratory activity compared to the GD2-populations along with high expression of MMPs (MMP7, and MMP19) and differential expression of EMT associated genes. GD2+ cells showed an increased expression of N-cadherin, Vimentin and a reduction in E-cadherin expression levels compared to the GD2-sub-population suggesting the direct involvement of GD2 in promoting pro-metastatic ability of the cancer cells (Battula et al., 2012). In a similar study with high grade bladder cancers (BLCA), expression of GD2 was correlated with the high grade of the cancer. Overexpression in the GD2 level in a subset of high-grade bladder cancer cell lines (UMUC3 and J82) was associated with higher migratory activity. Differential gene expression study among these cell populations showed an elevated expression of EMT associated N-cadherin, Vimentin. The result was further validated in the patient tissues, where high grade BLCA samples having overexpression of GD2, showed an elevated expression of the mesenchymal genes, identifying the pro-metastatic role of GD2 in BLCA(38). Gangliosides GM2 and GM1 were also shown to regulate migration and invasion in cancer cell lines of various origin. Overexpression of GM2 either by ectopically expressing GM2-synthase or by exogenous GM2 administration, was associated with increased cellular migration and invasion in the several cancer cell lines. Further study to identify the mechanism behind GM2 mediated activation of cellular pro-migratory properties identified that GM2 interacts with integrin-β1 receptor and modulates downstream signalling cascade by promoting activation of FAK, Src kinases to activate ERK-MAPK pathway. Activation of ERK-MAPK signalling pathway is well reported to be associated with increased tumorigenesis. GM2 further perturbed the G-actin/F-actin homeostasis and promoted stress fibre formation to promote the migratory property of the cancer cells. The observation was revalidated with a knockdown system, where knocking down GM2 from the cell lines showed formation of the stress-fibres which leads to reduced migration and invasion *ex-vivo* (Kundu et al., 2016). In mouse Lewis lung cancer cell line, reduced GM1 expression was associated with the higher metastatic and invasive property of the cells. Variants of Lewis lung cancer cell line with higher metastatic and proliferative activity showed a reduced GM1 expression while the expression of other gangliosides remains unchanged. It was further showed that reduced expression of GM1 is associated with enrichment of glycolipid containing microdomains (lipid rafts) in the plasma membrane which leads to the increased expression of integrin β1 receptor on the membrane rafts. Furthermore, low GM1 expression subsequently promoted the localization of MMP-9 to the membrane and formed a complex with integrin β1. The complex formation thus, activated and promoted MMP-9 secretion in low GM1 expressing variant of mouse Lewis’s lung cancer cell line resulting in higher metastatic and invasive activity (Zhang et al., 2006). The role of GM3 was also studied in a murine model of breast cancer. 4T1, a highly metastatic variant of breast cancer cell line showed an elevated expression of GM3. Knocking down GM3 in 4T1 cells showed a reduced migration and invasive properties of the cells *in vitro* and *in vivo*. Further analysis to identify the signalling mechanism revealed that knocking down GM3-synthase led to suppression of PTEN activity, an upstream regulator of Akt pathway. Suppression in the PTEN activity subsequently promoted Akt phosphorylation and reduced expression of NFAT1 (Nuclear factor of activated T cells). Reduced activity of transcription factor NFAT1 explains the molecular events behind the reduced tumorigenesis in GM3 synthase knocked down cells (Gu et al., 2008). The role of GD3 in regulating metastasis and invasion was extensively investigated in cancer (Liu et al., 2018). Initial studies with rat glioma C6 cell lines, stable overexpression of ST8Sia-I (enzyme that converts GM3 to GD3 endogenously) showed high proliferation rate along with elevated migratory and invasive property *in vitro*, clearly identifying the pro-migratory role of GD3 (Sottocornola et al., 1998). In malignant melanoma, overexpression of GD3-synthase in SK-MEL-28-N1cell line increased the invasive and migratory activity *in vitro*. Further study identified that high GD3 expression was associated with higher expression of Paxillin, a focal adhesion adapter protein that acts as a scaffold to form protein complex facilitating cellular migration and invasion. The higher expression of Paxillin was associated with GD3 mediated elevated migratory activity in malignant melanoma cell lines (Hamamura et al., 2005). Functional role of GD3-synthase gene was also studied in triple negative breast cancer (TNBC), an aggressive subtype of breast cancer with poor prognosis. Overexpression of GD3-synthase in MCF7 shows an increased migration, invasion and colony forming ability whereas knocking down GD3-synthase in MDA-MB-468 cell line, which normally showed high GD3-synthase expression, significantly reduced it. Further, identification of the mechanism behind the overexpression of GD3-synthase gene in TNBCs showed an interesting epigenetic regulation both *in vivo* and *in vitro*. The promoter of GD3-synthase gene in TNBC tissues as well as in the cell lines showed a hypomethylated state that led to an increased expression of the gene. The high expression of GD3-synthase was associated with lower relapse free survival and lower overall survival in TNBC patients (Li et al., 2019). Though studies have shown a positive effect of GD3-synthase on invasion and migration of cancer cells, a study on ER-negative breast cancer cell line MDA-MB-231 and SK-BR 3 revealed the ability of GD3 synthase to suppress the invasive potential by downregulating the expression level of ICAM-1. Stable overexpression of GD3-synthase gene in ER-breast cancer cell lines showed a selective upregulation of GD2 expression level. The overexpression supressed the migratory and invasive capabilities of the cells *in vitro*. ICAM1 downregulation upon GD3-synthase overexpression in ER-breast cancer but not in ER + cell line (MCF-7) suggested an association of estrogen receptor in GD2 mediated tumorigenesis. Promoter study of ICAM1 gene in GD3 synthase overexpressed condition showed a supressed transcriptional activity in ER-breast cancer cell lines (Kwon et al., 2017). Further study is required to identify the transcription factors hindering the transcriptional activity of ICAM1 gene in GD3 synthase overexpressed conditions. Recent research involving extracellular vesicles (EVs) has not only established their critical role in governing cellular communication but also highlighted the significance of glycolipids as a key structural component of EVs. Investigations into the lipid composition of extracellular vesicles (EVs) have revealed a notable enrichment of various gangliosides on the EVs’ outer membranes. Notably, this composition significantly differs from the ganglioside makeup of the parent cells and varies under different pathological conditions, including cancer (Rani et al., 2023). Overexpression of ST8Sia I (GD3 synthase) in normal murine melanoma cell line, Melan-a led to the conversion of GM3 into GD3, resulting in a reduction of GM3 levels and accumulation of GD3 in the cells. As a consequence of GD3 overexpression, the melanocytes displayed increased adhesion and greater migratory capabilities compared to their non-modified counterparts. Furthermore, extracellular vesicles secreted by the GD3 overexpressing cells showed an enrichment of GD3 on their membrane. Upon co-culture with the parental melanocytes, these vesicles induced a migratory response in the recipient cells. Subsequent investigations to demonstrate the mechanism identified that the melanocytes with GD3 overexpression secreted extracellular vesicles that were taken up by the normal Melan-a cells, leading to an increase in GD3 expression levels in the recipient cells, which promoted the pro-migratory behavior in the recipient cells. Conversely, when GD3-overexpressing Melan-a cells were exposed to extracellular vesicles from normal Melan-a cells (which had higher GM3 levels), the migratory activity of the GD3-expressing cells was significantly inhibited. This finding further supported the anti-tumor role of GM3. Therefore, the study established that tumor associated gangliosides carried by extracellular vesicles, could alter normal cellular behavior and influence tumorigenesis (Otake et al., 2019). In a comparable investigation, human melanoma cells of the SK-MEL-28 cell line were genetically modified by introducing ST8SIA1 and B4GALNT1 genes to overexpress GD2. The study aimed to examine how extracellular vesicles (EVs) originating from melanoma cells with elevated GD2 levels affected the characteristics of unaltered SK-MEL-28 cells. When control SK-MEL-28 cells were exposed to the EVs secreted by GD2-positive cells, several notable effects were observed. These effects included an increase in cellular growth, heightened invasiveness, and enhanced migratory properties in the recipient cells. Additionally, exosomes derived from GD2-positive cells promoted the rapid phosphorylation of various signaling molecules, including EGFR and FAK, and promoted their activation. The activation of these pro-tumorigenic signaling pathways provided further evidence of the cancer-promoting role of GD2 found within the secretory vesicles of these cancer cells, as it appeared to transform nearby normal cells and exacerbate the tumorigenic process (Yesmin et al., 2023). In another study using the MCF-7 breast cancer cell line and the normal breast epithelial cell line MCF10A, the overexpression of B3GALT4, the enzyme responsible for GM1 synthesis, led to increased GM1 expression and elevated EV secretion into the extracellular environment. This observation suggested the role of ganglioside GM1 in the cellular secretion of extracellular vesicles. It was further observed that overexpression of GM1 was associated with a substantial enrichment of GM1 on the outer surface of the secreted EV membranes. Interestingly, when wild type normal epithelial MCF10A cells were exposed to these GM1-enriched EVs, their migratory potential significantly increased, and they acquired more mesenchymal characteristics. Subsequent investigation revealed that these GM1-enriched EVs from cancer cells transferred their ganglioside components to recipient cells, promoting epithelial-mesenchymal transition (EMT) properties. This further upregulated mesenchymal-related genes such as vimentin and N-cadherins while downregulating the expression of E-cadherin, providing further insight into the molecular mechanism of EMT induction mediated by these GM1-enriched EVs in normal recipient cells (Ma et al., 2022). This suggests a novel and crucial regulatory role of EV associated gangliosides that orchestrates the cellular communication in different pathophysiological circumstances. These findings together show the importance of cellular gangliosides in regulating this hallmark of cancer. Table 1 summarizes the differential role of gangliosides on tumor migration and metastasis.

**TABLE 1:** Effect of different gangliosides on migration and invasion.

Name of ganglioside	Type of cancer	Effect on migration and invasion	Ref
GD2	Bladder Cancer	+	Vantaku et al. (2017)
GM2	Human Renal Cell Carcinoma, Glioblastoma, Pancreatic Ductal	+	Kundu et al. (2016), Sasaki et al. (2019)
GM1	Lewis lung Cancer	−	Zhang et al. (2006)
GD3	Triple negative Breast Cancer	−	Kwon et al. (2017)
	Melanoma, Breast Cancer	+	Hamamura et al. (2005), Li et al. (2019)
GM3	Breast Cancer	+	Gu et al. (2008)

## 6 Role in evading immune response

**FIGURE 5:**
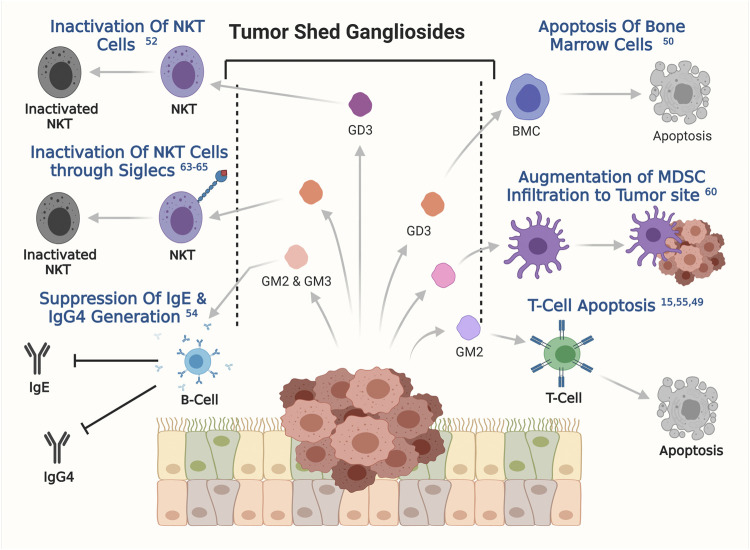
The involvement of tumor shed gangliosides in evading immune response. Involvements of specific gangliosides, obtained from the published reports has been depicted. The respective references are shown on the respective effects as superscripts.

Survival of cancer cells inside the human body and their fate in long-term development into full blown tumor, depends on how efficiently the cancer cells were able to evade the immune system of the host organism. The role of gangliosides in helping the cancer cells to evade host immune response has been a topic of interest for a long time. Interestingly, gangliosides were first identified as a factor to influence immune response in certain cancers, however their further association in other aspects of tumorigenesis were investigated later. In the late 90s, the study with brain derived gangliosides demonstrated their immune suppressive role by inhibiting the proliferation of activated T cells (Irani et al., 1996). Tumor-derived gangliosides blocked IL2 and IFN-γ transcription in activated T cells followed by inactivation of NF-κβ signalling pathways. Inactivation of NF-κβ signalling is associated with reduced phosphorylation of retinoblastoma gene, leading to cell cycle arrest. Though the immune suppressive effect of secreted ganglioside was tested back then but their effect in tumorigenesis was unknown until shown in a groundbreaking study by Robert Mc Kallip et al. They assessed the role of ganglioside in antitumor immune response in FBL-3 murine tumor model, where tumor derived gangliosides prevented the generation of tumor specific T cell response and enabled the tumor cells to escape host immune surveillance, a first line of host defence against the tumor growth. This report pointed towards the role of gangliosides in immune suppression to promote tumorigenesis (Irani et al., 1996; McKallip et al., 1999). Such phenomena was initially also reported in renal cell carcinoma (RCC), whereby explants from RCC patients were shown to have higher apoptogenic activity in primary T cells when compared with samples from healthy individuals (Uzzo et al., 1999). In line with this finding, another study showed that gangliosides derived from T cell lymphoma induced apoptogenic signaling in bone marrow cells, primarily through tumor shed GD3-mediated upregulation of the NF-κB/p53 axis and consequent apoptosis (Bharti and Singh, 2000). It has been also shown that in T cell lymphoma tumor shed gangliosides could inactivate NKT cells through their interaction with CD1d (Sriram et al., 2002). The same phenomenon was observed in ovarian cancer where GD3 showed the capability of inactivating NKT cells through their affinity towards CD1d, which acts as a stimulus for NKT cells inactivation (Webb et al., 2012). In glioblastoma multiforme (GBM), tumor shed gangliosides was reported to promote local or systemic host immune suppression by inducing T-cell apoptosis (Chahlavi et al., 2005). Later studies further identified the molecular mechanism where, tumor shed ganglioside GM2 from GBM cell lines was also shown to be involved in inducing the apoptosis in T cells through TNFR1 mediated activation of caspase cascade (Mahata et al., 2015b). Similarly, in RCC patients the higher expression levels of GM2 sensitized the tumor infiltrated T cells to apoptosis when compared to normal donor T cells (Biswas et al., 2009). Furthermore, RCC tumor derived gangliosides induces T cell apoptosis by suppressing INF-γ and IL-4 production from infiltrating T cells and altering mitochondrial permeability transition (MPT), this phenomenon is significantly blocked by GM2 specific Anti-GM2 antibody (DMF10.167.4) (Biswas et al., 2006). This finding is in conformity with the observation that secretion of TNF-α by infiltrating inflammatory cells in RCC, promoted generation, accumulation, and shedding of ganglioside GM2, thereby promoting T cell apoptosis (Raval et al., 2007). In addition to T cells, B cell function was also shown to be compromised by tumor shed gangliosides GM2 and GM3 (Kimata and Yoshida, 1994). Gangliosides GM2 and GM3 were shown to suppress the generation of IgE and IgG4 upon stimulation by IL-4 and IL-13 by inhibiting endogenous TNF-α production. Secretion and binding of TNF-α to their specific receptor on B cells is known to stimulate antibody generation and class switching (Kimata, 1995). In one of the pioneering works on ganglioside mediated regulation of dendritic cells, co-incubation of murine bone marrow progenitors or human CD34^+^ progenitor cells with ganglioside secreting neuroblastoma cells, resulted in a substantial inhibition of dendritic cell generation (Dendropoiesis) *in vitro* by up to 90%. However, pretreatment of neuroblastoma cells with a glucosylceramide synthase inhibitor, DL-threo-1-phenyl-2-decanolylamine-3-morpholino-1-propanol HCl (D-PDMP), at a concentration of 10 µM hampers ganglioside synthesis, and represses the ability of neuroblastoma cells to inhibit dendritic cell generation. Further studies identified the enrichment of GD2 and GM3 within the neuroblastoma derived tumor shed glycolipid components. Interestingly, tumor shed GD2 and GM3 together inhibited the generation of dendritic cells from bone marrow progenitor cells but only GM3 was shown to hamper the antigen presenting property of these dendritic cells in neuroblastoma. Reduction in the antigen presentation by dendritic cells led to poor T-cell mediated immune response against tumor derived antigens, which further assisted in the progression of tumorigenesis (Shurin et al., 2001). An *in vitro* study demonstrated that tumor shed gangliosides augment the infiltration of myeloid derived suppressor cells (MDSCs) at the tumor sites and impedes host anti-tumor immune responses. However, the intricate mechanism behind ganglioside mediated MDSC infiltration is yet to be clearly understood (Wondimu et al., 2014). In addition to this, sialic acid–binding immunoglobulin-like lectins or Siglecs play a very important role in ganglioside mediated immune evasion in cancer. Siglecs are immunoglobulin like lectins which are present on the surface of various immune cells and bind with gangliosides and sialoglycoproteins (Schnaar, 2023). In humans 15 such siglec molecules are reported, among which siglec-7 and siglec-1 are known to orchestrate the intricate networks of ganglioside mediated immune evasion in cancer. Siglec-7 is generally expressed on natural killer, myeloid and dendritic cells (Chan and Chan, 2022). The glycans present on the surface of cancer cells were shown to interact with siglec-7 on the surface of Natural killer cells leading to immune checkpoint inhibition and subsequent immune evasion (Nicoll et al., 1999; Hugonnet et al., 2021). Evidence from neuroblastoma proved cancer cell derived disialogangliosides could also cause immune evasion through binding with siglec-7. Treatment with anti GD2 antibody was also shown to sensitise human cancer cells to macrophage mediated phagocytosis by blocking siglec-7 binding (Theruvath et al., 2022), clearly suggesting the role of GD2 in Siglec-7 mediated immune evasion in cancer. Siglec-1 on the other hand was known to be expressed in human macrophages and interacts with GM3, GD1a, GD1b, GT1b resulting in phagocytosis (van der Haar Àvila et al., 2023). Taken together, these findings have solidified the importance of tumor shed gangliosides in helping cancer cells to evade and escape the host immune response, thereby promoting survival, growth, and overall progression of the disease. A graphical overview of the modulatory role of ganglioside in host immune response is presented in Figure 5.

## 7 Gangliosides and angiogenesis

Angiogenesis, the process that depends on complex signaling machinery primarily mediated by growth factors such as VEGF, leading to formation of blood vessels is one of the crucial phenomena dictating the development and sustainability of tumors. Over the last few years, several pivotal studies have implicated angiogenesis to be regulated by endogenous or tumor shed gangliosides. This information was generated from the observation that GD1a enhances VEGF’s ability to induce angiogenesis in HUVEC cells (Lang et al., 2001). This initial finding is also supported by studies that were published later, where ganglioside deficient murine tumor cells (DKO cells) gave rise to almost avascular tumors when compared to tumors derived from ganglioside rich wild type cells, indicating a regulatory role of gangliosides in angiogenesis (Liu et al., 2014). Similar to GD1a, some other gangliosides like GM1 and GM2 are also shown to have pro-angiogenic roles. While GM2 was shown to induce angiogenesis by inducing CD31 positive endothelial cells, enhancing VEGF and inducing endothelial cell proliferation, GM1 was shown to induce angiogenesis through MCP-1 receptor (Chung et al., 2017; Yoshida et al., 2020). GM3 on the other hand was shown to act as an anti-angiogenic factor by several groups (Chung et al., 2009; van Cruijsen et al., 2009; Seyfried and Mukherjee, 2010). A detailed study on GM3 mediated suppression of angiogenesis revealed that GM3 physically interacts with VEGFR2, therefore blocking its dimerization (Chung et al., 2009) resulting in suppressed angiogenesis. This is also supported by other observations, that high GM3 levels are associated with low vascularization in both mouse astrocytoma tumors (Seyfried and Mukherjee, 2010) and human Non-Small Cell Lung Cancer (NSCLC) patient samples (van Cruijsen et al., 2009). Although the studies on the involvement of gangliosides in regulating angiogenesis are relatively few, the generated information carries immense importance in understanding how tumor shed factors influence its surrounding cells.

## 8 Ganglioside and apoptosis

**FIGURE 6:**
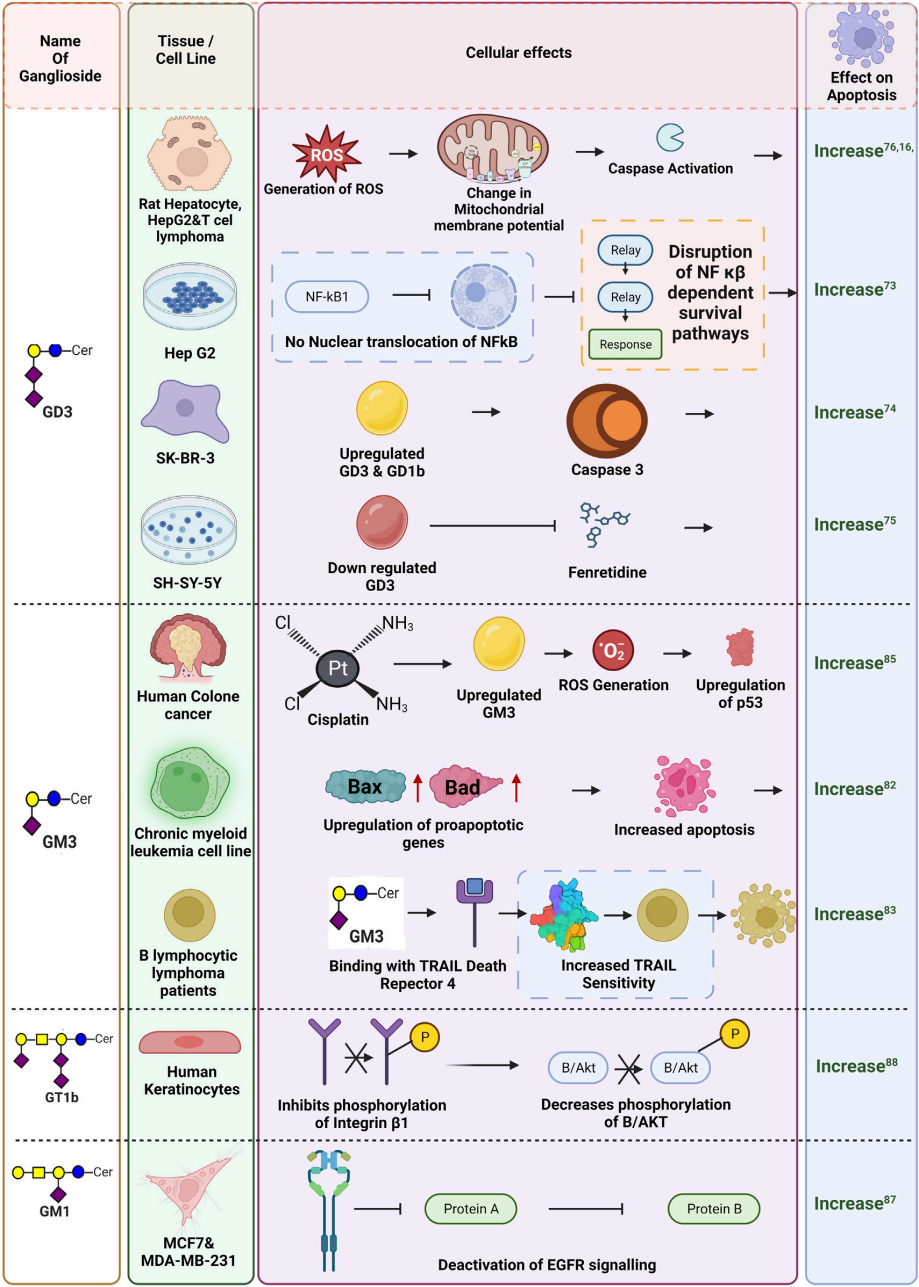
Differential impact of gangliosides in apoptosis. Influence of gangliosides on different pathways leading to apoptosis are shown here. Gangliosides are reported to both promote and suppress apoptosis by influencing different pathways as depicted in this figure. The respective references are given as superscripts.

Normal cells maintain intricate balance between cell survival and cell death pathways to maintain cellular homeostasis. This homeostasis is perturbed in cancer cells, which leads to inhibition of apoptotic pathways, and promotion of cell growth and survival pathways, leading to uncontrolled cellular proliferation. Cancer cells generally overcome cellular death by activating different anti-apoptotic pathways. Evidence suggest the differential involvement of gangliosides, especially GD3 in by-passing apoptogenic signaling in ROS-dependent manner in rat hepatocytes and human HepG2 cells (Colell et al., 2001). GD3 promotes the generation of ROS leading to mitochondrial permeability transition (MPT) and elicit cytochrome-c release and caspase activation. Furthermore, GD3 abrogates the nuclear translocation of NF-κB and its downstream survival pathways in rat hepatocytes and HepG2 cell line. Thus, perturbation of the NF-kB dependent survival pathway by GD3 sensitize the cells to apoptosis-inducing stimuli. In HEPG2 cells, treatment with ionizing radiation or daunorubicin leads to upregulation of NF-κB dependent genes which is accompanied by minimal killing of the cells. However, pre-treatment of GD3 prevents the translocation of NF-κB family members and suppresses NF-κB dependent gene induction leading to higher cell death upon daunorubicin treatment (Paris et al., 2002). Apoptotic activity of GD3 is also reported where exogenous administration of ganglioside GD3 and GD1b was found to promote apoptosis in the human breast cancer cell line (SK-BR-3) in a caspase 3 dependent manner (Ma et al., 2004). Furthermore, in neuroblastoma cell line SH-SY5Y, downregulation of GD3 synthase abrogates Fenretinide (a synthetic derivative of retinoic acid) induced apoptosis in neuroblastoma by disruption of GD3- 12-lypoxygenase axis (Lovat et al., 2005). An increase in the endogenous GD3 level by the overexpression of GD3 synthase changes the mitochondrial membrane potential and activates apoptotic signaling in T cell lymphoma, by promoting the release of cytochrome-c. Caspase9 dependent apoptosis and suppression of Bcl2 is also reported in case of T cell Lymphoma (Rippo et al., 2000). In tumorigenesis, the involvement of GD3 in promoting apoptotic signaling is well documented. In a study with a human lymphoid cell line CEM, GD3 was reported to promote FAS-mediated apoptosis. The study showed that triggering FAS either by FAS ligand or anti-FAS antibody led to GD3 accumulation in the apical region of CEM cells where it colocalized with Ezrin protein to promote FAS-dependent apoptosis. It was further shown that either pharmacological inhibition of GD3 or siRNA-mediated knockdown of Ezrin blocked Fas-dependent apoptosis in CEM cells, clearly suggesting the involvement of ganglioside GD3 in Fas-mediated activation of apoptotic signaling (Giammarioli et al., 2001). The same group also illustrated a detailed mechanism in support of the previous observation. In CEM cells, triggering Fas not only rearranged and redistributed GD3 to the apical region of the cells but also promoted its interaction with the CLIPR-59 protein. CLIPR-59 is a microtubule binding protein with a lipid raft binding domain, that transports GD3 to the mitochondria. In mitochondria, GD3 interacted with mitochondrial permeability transition pore complex to change membrane permeability, enhanced ROS production, and cytochrome-c release to activate caspase cascade to promote apoptosis (Sorice et al., 2010). Surprisingly, a post-synthetic modification leading to the addition of an acetyl group to the terminal sialic acid residue to form 9-O-acetyl GD3, which is aberrantly overexpressed only in tumor tissues, exhibited an anti-apoptotic property, unlike GD3. A study with glioblastoma tissues identified an overexpression of 9-O-acetyl GD3 compared to GD3. The study was further validated with glioblastoma cell lines where accumulation of 9-O-acetyl GD3 was shown to inhibit FAS-dependent apoptosis. Interestingly, cleaving the acetylated region of GD3 by treating the cells with hemagglutinin esterase, an enzyme that specifically cleaves the 9-O-Ac group of the 9-O-acetyl GD3, reversed its anti-apoptotic activity. It was further shown that the acetyl group in 9-O-acetyl GD3 blocked its binding to the mitochondrial permeability transition pore complex in Fas-triggered cells and failed to elicit mitochondria-dependent apoptotic signaling. This clearly explained the anti-apoptotic activity of 9-O-acetyl GD3 in tumorigenesis. Thus, Ac-GD3: GD3 ratio is a crucial determinant of cellular survival in glioblastoma (Birks et al., 2011). An *in vitro* study in neuroblastoma cell line NBL-W shows higher apoptotic activity in the presence of GM1, GM3, GD1A and GT1B (Mirkin et al., 2002). Exogenous administration of GM3 promotes cellular apoptosis in human and rat glioma cell lines (Fujimoto et al., 2005) while, apoptotic activity of GM3 is also well reported in human bladder cancer, which is more prevalent in superficial tumors than in the more invasive ones. Generation of a GM3 rich ganglioside pattern was also reported to increase the propensity of the cells to undergo apoptosis in chronic myeloid leukemia cell line K562 via the increase of mRNA levels of pro apoptotic proteins like Bax and Bad and by affecting the balance of pro and anti-apoptotic proteins inside the cells (Tringali et al., 2009). Also, in B-chronic lymphocytic lymphoma patients, GM3 was shown to associate with TRAIL death receptor DR4 therefore, increasing the cells susceptibility to TRAIL (Tumor necrosis factor-related apoptosis-inducing ligand) followed by subsequent apoptosis. This GM3-DR4 interaction was found to be negligible in B-cells from healthy donors (Marconi et al., 2013). Another study showed GM3 to induce sensitivity of EGFR-TK blocker type chemotherapeutic drugs in 3LL lung cancer cell lines by increasing EGFR protein levels (Noguchi et al., 2007). Surprisingly, overexpression of GM3 synthase in GM3 deficient murine bladder carcinoma cell line MBT-2 results in more apoptotic cells *in vitro* (Watanabe et al., 2002). Cisplatin, a well-known chemotherapeutic drug, promoted cellular death by up-regulating GM3 synthase expression and thereby up-regulating ganglioside GM3 in human colon cancer. This was confirmed by both siRNA mediated downregulation of GM3 synthase as well as ectopic overexpression of GM3 synthase in HCT116 cell line (Chung et al., 2014). Conversely, overexpression of GM3 synthase in 3LL Lewis lung carcinoma cell line desensitizes the cells from doxorubicin and etoposide mediated apoptosis by blocking caspase activity and Bcl2 upregulation (Noguchi et al., 2006). Another well-known ganglioside GM1 is reported to be overexpressed in breast cancer, which can impair contact-dependent growth in MCF7 and MDA-MB 231 cell lines by deactivation of EGFR signaling (Zhuo and Guan, 2019). A study on GT1b suggested its interacting ability with integrin α5β1 to induce apoptosis in keratinocytes by promoting cell cycle arrest (Wang et al., 2001). It was further shown that GT1b induced apoptosis in keratinocytes by inhibiting the serine/threonine phosphorylation of integrin β1 as well as by decreasing phosphorylation of integrin linked kinase B/Akt at Ser-473 site therefore activating caspase 9. Figure 6 represents the differential regulation of gangliosides in apoptosis.

## 9 Targeting gangliosides through immune therapy

**FIGURE 7:**
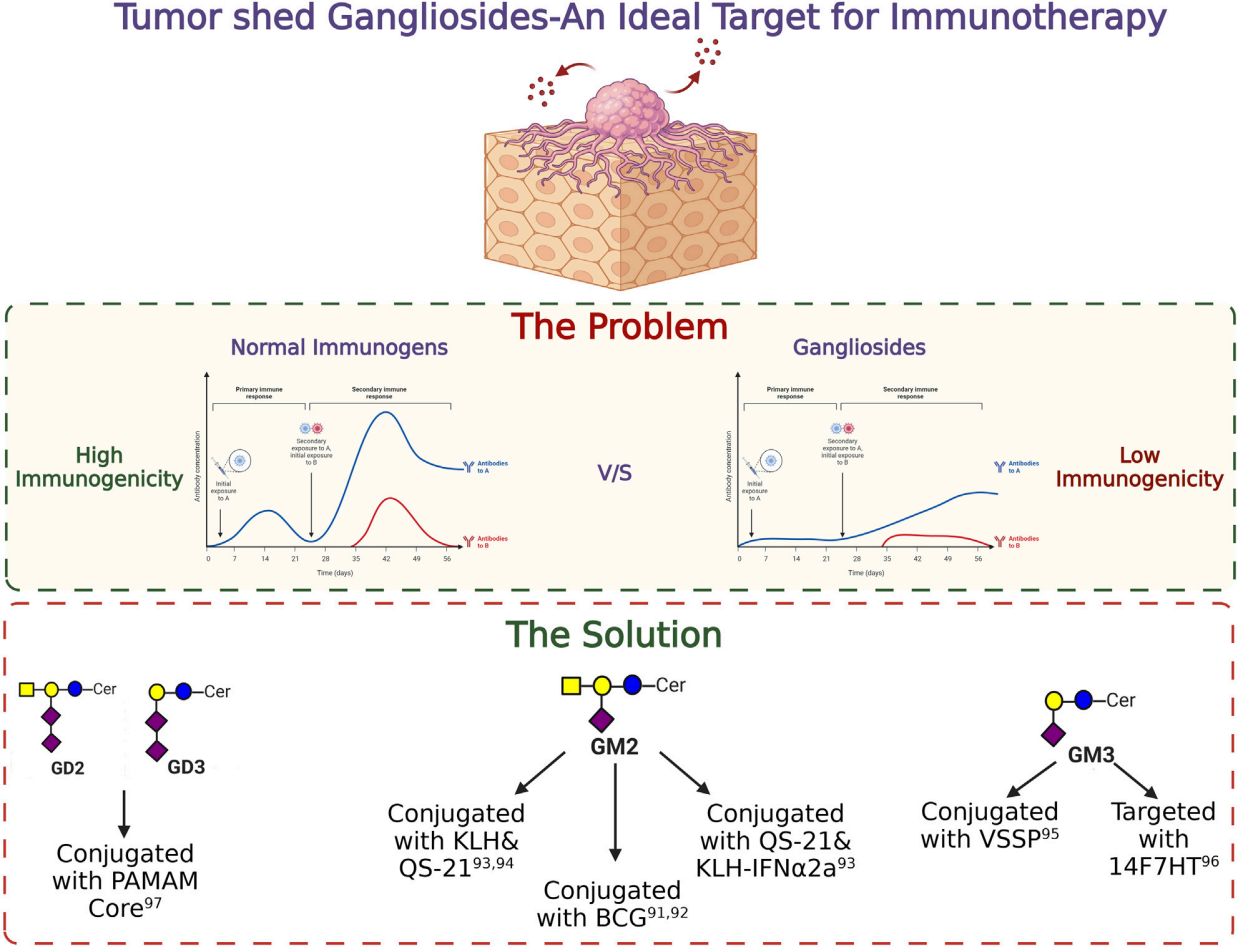
Targeting gangliosides through immunotherapy. The importance of tumor shed gangliosides and the strategies to target them are depicted in this figure.

Differential expressions of gangliosides are well studied in various cancers. Advancement in the field of glycobiology identified gangliosides as a potent target for future immunotherapy. Due to their aberrant overexpression in cancer cells in comparison with normal cells, they provide a unique opportunity for selective treatment without hampering the functioning of the normal cells (Daniotti et al., 2013). However, the poor immunogenicity of ganglioside molecules makes it difficult to generate antibodies during the initial days of the study. A pioneering work by Friedhelm Helling in the field of ganglioside vaccine development involved the conjugation of carrier proteins to the ganglioside moiety thereby generating a humoral immune response. Apart from the effective generation of antibodies against specific gangliosides, different adjuvant conjugated vaccines were used to overcome poor antigenic properties of gangliosides and to successfully elicit a strong cell-mediated immune response *in vivo*. Administration of BCG/GM2 conjugates efficiently generated GM2 specific antibodies in melanoma stage 3 patients, resulting in significantly longer disease-free state and prolonged survival (Helling and Livingston, 1994). Ritter et al. in a groundbreaking study identified ganglioside as a potent immunogen and successfully generated antibodies against GM2 which was further tested in several clinical trials (Livingston et al., 1994; Kitamura et al., 1995). In a clinical study, among one hundred twenty-two patients with stage III melanoma, 58 patients received GM2/BCG vaccine, and 64 received BCG alone. All patients were treated with low-dose cyclophosphamide before immunization. Patients with successful production of antibodies against GM2 within GM2/BCG groups showed significantly longer disease-free interval than that of the BCG group and a longer overall survival was also observed among the GM2/BCG group patients. But the side effects during the study were also recorded. When BCG was administered intradermally, all patients who had received three or more immunisations showed signs of inflammation and eschar development with drainage. During the following immunisations the dosage of BCG was lowered in order to manage the inflammatory response. Surprisingly, GM2 had no impact on the inflammatory response to BCG and did not result in additional side effects. Adjuvants like saponin such as QS-21 was further used to conjugate gangliosides along with carrier proteins like KLH to enhance the immunogenicity. Conjugating GM2 with a carrier protein KLH and QS-21 was able to generate a higher immune response with IgG (71%) and IgM (88%) against GM2 (Chapman et al., 2000). In another phase II clinical study with 107 melanoma patients, administration of GM2-KLH/QS-21 in combination with IFNα2b significantly improved the antibody response which generated 90% IgM and more than 92% IgG response against GM2. During the trial, multiple cases of grade 3 and grade 4 toxicity were identified, but no treatment-related deaths were noted. Five individuals who received the combined therapy exhibited grade 4 toxicity. Of the 41 patients, the most prevalent severe (grade 3) adverse effects were tiredness, granulocytopenia, and raised liver enzyme levels. However, 66 people, including 60 patients who received the combinational therapy, experienced more severe grade 3 and higher side effects (Kirkwood et al., 2001). With further advancement in the field of antibody generation, NeuGcGM3 was combined with the outer membrane protein complex of *Neisseria* meningitidis to produce proteo-liposomes (VSSP), which resulted in successful development of antibodies against GM3 in breast cancer patients in a phase I clinical study. There were few side-effects reported during the study. Short-term pain at the injection site and local skin responses, such as induration, erythema, soreness, and oedema, were the most common side effects among the patients. Additionally, a few patients experienced fever, chills, myalgias, arthralgias which were disappeared by usual antipyretic treatment (Carr et al., 2003). In another similar study using humanized antibody 14F7hT, targeting all the variants of ganglioside GM3 (Neu5Gc, Neu5Ac) exerted antitumor effects *in vivo* by exerting antibody-dependent cell-mediated cytotoxicity (ADCC) (Dorvignit et al., 2019). Conjugation of PAMAM core with ganglioside GD3 and GD2 is also known to increase the immunogenicity of the gangliosides (Tong et al., 2019). Moreover, the transfer of adaptive T cells from vaccinated mice significantly reduced the tumor volume in non-vaccinated mice, clearly raising the hope for using ganglioside specific antibody-mediated immunotherapeutic strategy for treating cancer. The success of anti-ganglioside Ab therapy will ultimately depend on successful and accurate characterization of the ganglioside(s) in question, and in combination with targeted chemotherapy for cancer patients. The basic strategies to target different gangliosides for immunotherapy in cancer has been illustrated in Figure 7.

## 10 Utilizing gangliosides as immuno-therapeutic targets in cancer

Therapeutic approaches in cancer by targeting gangliosides generally include using either specific chemotherapeutic agents targeted towards specific gangliosides or selective activation of an immune network against gangliosides using active or passive immunization. Minnelide, an analog of Triptolide (Noel et al., 2019), an epoxy diterpene lactone, and the only reported GD3S inhibitor (an enzyme responsible for the biosynthesis of GD3 and GD2), has been used as a chemo-therapeutic agent against specific ganglioside. It is given either alone or in combination with Paclitaxel in an ongoing phase I clinical trials among the patients with refractory gastric cancer and advanced solid tumors (NCT03129139). In an ongoing phase-II clinical study among Adeno-squamous cell carcinoma of the pancreas (ASCP) patients, Minnelide is used as a GD3S blocker to therapeutically target ASCP, a rare form of pancreatic cancer with limited chemotherapeutic availability (Skorupan et al., 2022). Passive immunization with murine or murine/human chimeric monoclonal antibodies targeting gangliosides in their native or modified form in association with other effector molecules has been studied extensively. One of the fundamental aspects of immunotherapy is the localization of the immune effector molecules at the site of the lesion. During the early days of clinical studies, iodine labeled 3F8, and MabR24 targeting GD2 (Grant et al., 1996), and GD3 (Vadhan-Raj et al., 1988; Casper et al., 1994) respectively, showed successful localization at the tumor sites, which encouraged further investigation in this field. Most of the clinical trials with gangliosides were reported in malignant melanoma, neuroblastoma, and in small cell lung cancer (SCLC) patients. Trials with antibody driven targeting of GD3 and GD2 resulted in partial remission and prolonged survival in some cases, however, these treatments showed several side effects, for, e.g.,: toxicity, abdominal and pelvic pain (Saleh et al., 1992), pulmonary capillary leak (Kirkwood et al., 2000), pain and erythema at tumor sites (Scott et al., 2001), fever, deregulated urination, etc. Apart from passive immunization, a strategy using an anti-idiotypic antibody (anti-Id) against the endogenous antigen-specific antibody to activate cell-mediated immune response showed significantly better results than passive immunization and primary immunization with specific ganglioside. These anti-Id antibodies in combination with adjuvants proved to be more successful with much lesser toxicity (Fredman et al., 2003). In melanoma patients, immunization with BEC2 (anti-Id-GD3) with QS-21 resulted in an enhanced anti- GD3 response. After following the treatment for 2 years, more than 60% of patients were disease free (McCaffery et al., 1996). Apart from melanoma, the results using ganglioside-dependent immunotherapy in other cancers are inconsistent with mild to severe side effects. However, a combinatorial approach using different cytokines and chemokines with Mab R24(targeting GD3), Mab ch14.18 (targeting GD2), and Mab 3F8(targeting GD2) showed a longer survival with some level of side effects. In several clinical trials among multiple myeloma patients, Mab R24 along with different cytokines and chemokines result in severe side effects including Lymphocytosis, Monocytosis, Urticaria, Anaphylaxis (Bajorin et al., 1990; Minasian et al., 1995; Alpaugh et al., 1998). Prolonged relapse-free survival was seen in neuroblastoma patients participating in clinical trials with Mab ch14.18, but no appreciable clinical changes were seen in multiple myeloma patients. Numerous adverse symptoms remained in both cases, including urticaria, motor neuropathy, pruritus, and abdominal pelvic pain (Saleh et al., 1992; Handgretinger et al., 1995). In a phase II clinical trial among neuroblastoma patients, administration of Mab 3F8 in combination with GM-CSF resulted in complete remission in 17 patients among 45 participated patients. It also included the side effects such as fever, abdominal pain, hypotension, etc (Kushner et al., 2001). Dinutuximab (an anti-GD2 mAb), approved by US Food and Drug Administration (US-FDA) is currently used as a combination immunotherapeutic regimen to treat children with high-risk neuroblastoma (Greenwood and Foster, 2017). A phase-III clinical study (Clinical trial no: NCT00026312) with isotretinoin, cytokines alone or in combination with Dinutuximab in Neuroblastoma patients to assess the efficacy of Dinutuximab over conventional therapeutic regime demonstrated an overall improvement in the 3years event free survival in patients undergoing immunotherapy (78.8%) as compared to those undergoing conventional therapy (67.4%). Apart from the apparent advantage of immunotherapy over conventional therapy among neuroblastoma patients, several adverse side effects were also recorded, which included anemia, abdominal pain, diarrhea, fever, allergic reaction, anaphylaxis, several metabolic disorders like hypokalemia, hyponatremia, respiratory problems and other vascular disorders like capillary leak syndrome and hypotension.

Specificity and availability of the antigen are two most crucial bottlenecks for a successful immunotherapeutic approach. To overcome that, CAR-T cell-based therapies targeting specific antigen is an interesting and developing field in cancer immunotherapy. A CAR-T cell-based therapy employs T cells from cancer patients which were genetically modified to express chimeric antigen-specific receptors that target overexpressed antigens thereby targeting cancer cells while leaving the normal cells unharmed. Targeting specific ganglioside using CAR-T cells is a growing field towards glyco-immunotherapy. Initial clinical studies with GD2. CAR-T in neuroblastoma patients failed to achieve significant clinical response, but further modification of the CARs and inclusion of an IL-15 cassette (GD2. CAR.15) exhibited superior antitumor activity toward neuroblastoma by promoting enhanced tumor cell apoptosis and elevated CAR-T cell survival in the peripheral blood in a preclinical murine model (Chen et al., 2019). An ongoing phase-I clinical study to assess the dose and determine the side effects of CAR-T cells targeting GD2 in patients with refractory/relapsed osteosarcoma and neuroblastoma is under way (Clinical trial no: NCT04539366). Another phase I clinical study (Clinical trial no: NCT05620342) aims to investigate patients with small cell lung cancer (stage-IV) using autologous CAR-T (iC9-GD2. CAR.IL-15 T) cells targeting GD2.

The cytotoxic efficacy of GD2 targeting CAR-T cells in Retinoblastoma was also demonstrated in a preclinical model. The fourth generation GD2 targeting CAR-T cells have shown improved T cell generation and elevated cancer cell specific killing effect *in vitro*. But unfortunately, residual Retinoblastoma cells became resistant to these CAR-GD2 T cells by selective attenuated expression of GD2, and increased PD-L1 expression on tumor cells, suggesting a combinatorial approach along with the genetically modified T cells to achieve a more pronounced antitumor effect in Retinoblastoma. However, the prevalence of gangliosides on the normal tissues and nerve fibers, limited the comprehensive success of targeted therapy. An extensive study on tumor glycobiology leading to the identification of the O-acetyl derivatives of ganglioside GD2 and their specific localization on tumor tissues (Alvarez-Rueda et al., 2011)established O-acetyl GD2 as a tumor-associated antigen which can be further targeted for immunotherapies. mAb 8B6 that targets O-acetyl-GD2, provides a very promising safety reactivity profile during its clinical use but poor immunogenicity of O-acetyl-GD2 was a major concern.

## 11 Conclusion

This article delved into the diverse functions and intricate involvement that gangliosides play in the different aspects of tumorigenesis. Further, this review also focussed on the recent developments in the field of ganglioside biology and their direct implication in cancer immunotherapies either by directly targeting gangliosides or by modulating its downstream effectors in different cancers. Numerous studies highlighted the significance of gangliosides and raised the prospect of utilizing these glycolipids both as biomarkers, as well as potential therapeutic targets against various cancers. Among most gangliosides, the involvement of GD3 and GD2 has been investigated rigorously in the context of tumour initiation, development, progression, as well as an immunotherapeutic target against cancer. The role of GD3 and GD2 in the regulation of cellular proliferation, differentiation (Alvarez-Rueda et al., 2011), and evading immune checkpoint (Webb et al., 2012) which contributes to the invasion and metastasis of the tumor cells has been well established. GD3, typically a minor ganglioside in normal tissues exhibits a remarkable increase in different cancer cells. In melanoma, small cell lung cancers (SCLCs) (Malisan and Testi, 2002), and in patients with T-cell acute lymphoblastic leukemia (Furukawa et al., 2012) GD3 has been identified as a potential biomarker owing to its high expression compared to normal tissue. Similarly, GD2 shows a notably high expression in melanoma, small cell lung cancer (SCLCs) (Malisan and Testi, 2002), osteosarcoma (Poon et al., 2015), aggressive subtypes of breast cancer (Orsi et al., 2017) and neuroblastoma (Terzic et al., 2018) enables it to serve as a biomarker for these cancers. In addition to its significantly high expression levels in certain cancers, the ability of GD2 to maintain the stem cell phenotypes in breast cancer (Liang et al., 2017) was also implicated.

The importance of gangliosides as an impactful regulator of cellular function has increased significantly over the past few years following several studies indicating its capability to regulate the hallmarks of cancer directly or indirectly. Following initial studies that suggested such an involvement, further studies have solidified this link. Gangliosides are not only reported to be engaged in modulating several hallmarks such as migration and invasion, promoting proliferative signalling, evading host immune response, angiogenesis or avoiding cell death signals, but also their role in these regulations were reported to be multi-dimensional, and often context-dependent. Such contrasting dynamics in ganglioside behaviour have invited more and more studies which included attempts of targeting select gangliosides for immunotherapy, providing us with a scope of venturing effective and targeted therapeutic strategy against cancer. Despite promising results, several challenges remain in ganglioside-based anti-cancer therapeutic approaches. The necessity of anti-ganglioside conjugates, owing to poor immunogenicity of gangliosides, in general have been presented with moderate to severe side effects in several clinical trials, despite positive therapeutic achievements. Hence, the need for a targeted approach which will provide better specificity towards cancer cells. Interestingly, the absence of O-acetyl GD2 in normal tissues and peripheral nervous system reduces tumor off-target effects resulting in significantly reduced toxicity with O-acetyl-GD2 mAb 8B6 (Alvarez-Rueda et al., 2011). Constant efforts are therefore required for a better perception of the functional involvement of acetyl derivatives of gangliosides in tumor cells, since this information will provide a better understanding for the rational design of anti-O-acetyl-GD2 therapeutic antibodies. Comprehensive lipidomic studies focusing on the differential expression of various gangliosides in different tumor tissues and/or cell lines along with their corresponding normal uninvolved tissues and/or normal cells, may provide a holistic understanding of ganglioside expression in tumors and may also provide a differential ganglioside signature that differentiates tumor from non-tumour (Chen et al., 2016; Del Boccio et al., 2017). Furthermore, immunotherapeutic approach using CAR-T cells targeting gangliosides have shown great promise. Recently, several clinical trials with CAR-T cells targeting GD2 have been ongoing. Since, CARs can be modified with different co-stimulatory signalling molecules to generate robust host immune response against specific gangliosides, further exploration to utilize the CAR-T system is required to specifically target select gangliosides more efficiently. Identification of ganglioside interacting Siglecs and their role in immune regulation opens a new avenue towards targeted therapeutics. Targeted delivery of gangliosides to facilitate their interaction with Siglecs followed by immune activation may open future avenues for utilizing gangliosides to target cancer cells. Hence, the functional association between Siglecs and gangliosides need further studies to exploit their potential as a therapeutic target in cancer. A plethora of studies link gangliosides and their effects on the immune cells thereby highlighting them as critical instruments of tumor-induced host immune suppression. Recently, immune-checkpoint blockade has come up as efficient therapeutic strategies against many cancers, especially cancers of the blood. However, whether gangliosides might influence immune-checkpoints and if so, how, is completely unknown. A mechanistic understanding of this may provide a better insights into those subsets of cancers that are refractory to immune checkpoint blockade therapy. Overall, gangliosides not only have come up as a critical determinant of tumorigenicity and dictate tumour aggressiveness, but also have emerged as potential targets of therapeutic value. However, their complexities in function, arising mostly from the diversity of their structure, have created hindrance in fully understanding their biological role. With the advancement in mass spectrometric approaches like lipidomic, coupled with genomics and proteomics might pave the way towards their precise identification, differential profiling and functionalities which might help to utilize their full potential as crucial anti-cancer targets.
